# Bridging the Gap: Biological Reconstruction with Vascularised Fibula, Massive Allograft, or Capanna Technique After Intercalary Resection in Children and Young Adults with Lower Limb Bone Sarcoma

**DOI:** 10.3390/children13070952

**Published:** 2026-07-20

**Authors:** Lorenz H. M. van Schalkwijk, Maria Clara Correia, Whitney L. Kenswiel, H. Chien Nguyen, Michiel A. J. van de Sande, David D. Krijgh, Lizz van der Heijden

**Affiliations:** 1 Princess Máxima Center for Pediatric Oncology, 3584 CS Utrecht, The Netherlands; l.h.m.vanschalkwijk-5@prinsesmaximacentrum.nl (L.H.M.v.S.); m.a.j.vandesande-2@prinsesmaximacentrum.nl (M.A.J.v.d.S.); 2 3D Lab, University Medical Center Utrecht, 3584 CX Utrecht, The Netherlands; w.l.kenswiel@umcutrecht.nl (W.L.K.); h.c.nguyen-3@umcutrecht.nl (H.C.N.); 3 Department of Orthopedic Surgery, Leiden University Medical Center, 2333 ZA Leiden, The Netherlands; 4 Department of Plastic and Reconstructive Surgery, University Medical Center Utrecht, 3508 GA Utrecht, The Netherlands; d.d.krijgh-3@umcutrecht.nl

**Keywords:** primary bone tumours, limb salvage, bone allograft, vascularised fibula, biological reconstruction

## Abstract

**Highlights:**

Intercalary resection of malignant bone tumours results in bone defects that lead to reconstructive challenges in the lower extremity. In paediatric patients, biological methods for restoring these defects include the use of massive allografts, vascularised fibular grafts, or combined techniques (i.e., the Capanna technique). Although these methods are commonly used in paediatric oncology, literature analysing their surgical outcomes is still limited. This review outlines the current evidence on the outcomes of these biological methods in paediatric and young adult patients following intercalary resection for lower-extremity bone sarcoma.

**What are the main findings?**

- Massive allograft or the Capanna technique are viable options for large segmental bone defects in paediatric and young adult patients after lower-extremity bone sarcoma resection, offering reliable union, early weight-bearing, and low complication rates. Despite their avascular nature, massive allografts demonstrated the shortest time to union in this purely paediatric review.

**What is the implication of the main finding?**

- Reconstruction strategy for paediatric patients should be tailored to anatomical location, defect size, and patient-specific factors, including relative defect length rather than absolute thresholds. The Capanna technique may be particularly useful in paediatric patients when risk factors such as chemotherapy, poor host bone quality, or high mechanical load are present, although it is associated with donor-site morbidity, longer operative time, and requires microsurgical expertise.

**Abstract:**

**Background.** When free margins can be achieved and adjacent joints can be preserved, limb salvage surgery is the preferred treatment for intercalary paediatric bone tumours in the lower extremity. Biological reconstruction techniques include allograft, free vascularised fibular graft (FVFG), and allograft combined with FVGF (Capanna technique). This review summarises current evidence on outcomes and complications, and proposes directions for future research to refine patient selection. **Methods.** A systematic review was conducted using PubMed, Scopus, Embase, and Cochrane Library. Eligible studies reported outcomes of allograft, FVFG, or combined techniques for intercalary lower limb reconstruction in children or adolescents. **Results.** Twenty-five articles met the inclusion criteria (8 allograft, 7 FVFG, 14 Capanna); four studies reported two techniques. In total, 391 patients were included (allograft 147, FVFG 51, Capanna 193). Mean defect lengths were 14.9 cm (allograft), 14.5 cm (FVFG), and 14.7 cm (Capanna). Time to union was 8.5 months for allografts, 11.4 for Capanna, and 12.5 for FVFG. Non-union occurred in 14.7%, 13.2%, and 10.3%, and delayed union in 13.6%, 4.8%, and 7.9% for FVFG, allograft, and Capanna, respectively. Fractures were 40.5% for FVFG, 18.4% for allograft, and 23.5% for Capanna. Infections were least common with FVFG (2.7%). **Conclusions.** The reconstruction strategy should be tailored to anatomical location, defect size, and patient-specific factors. Although infections rates were lowest with FVFG, time to union was longer and the postoperative fracture rate was higher compared to the allograft or Capanna technique. Allografts demonstrated the shortest time to union and lowest fracture risk. The Capanna technique may be beneficial when factors including chemotherapy, poor host bone quality, or high mechanical load are present, but comes at a cost of morbidity and complexity.

## 1. Introduction

Limb salvage surgery (LSS) with reconstruction is currently the preferred surgical treatment for bone sarcomas of the lower extremity in children and young adults, when free margins can be achieved and when the adjacent joints can be preserved. It is often combined with (neo)adjuvant therapies, and offers better functional outcomes, while survival rates remain comparable to amputation [1,2].

Reconstructions of segmental bone defects remain challenging in paediatric patients, especially when the defects are longer present [3]. Moreover, in the paediatric population, it is important, when oncologically safe, to preserve the adjacent joint and epiphyseal plate of the affected limb to allow for remaining bone growth. Currently, there is a lack of literature analysing the surgical outcomes of biological reconstructions after intercalary resection in paediatric patients with a segmental resection due to a lower-extremity bone malignancy. Possible biological methods for restoring intercalary bone defects include a massive allograft, a free vascularised fibula graft (FVFG), or a combined technique with allograft and FVFG (i.e., Capanna technique or modification thereof, referred to hereafter as the Capanna technique in this review). An allograft is favourable on account of its strength and mechanical support and convenience as an “off-the-shelf product”. However, the avascular nature of the allograft is associated with complications such as non-union, graft fracture, and postoperative infections. Alternatively, reconstruction with an autologous vascularised fibula has the advantage of intrinsic blood supply that promotes bone formation. These enhanced vascular and osteogenic capabilities can facilitate primary bone healing, promote bone union, and improve structural integrity of the bone. Drawbacks of using a vascularised fibula graft include the risk of flap failure (failure of the microsurgical anastomosis), donor-site morbidity (including risk of peroneal nerve palsy), and autograft fractures, since a fibula shaft as standalone reconstruction usually does not provide enough structural stability when compared to massive allografts or a combination, especially in larger children and adolescents and in weight-bearing localisations [4]. In clinical practice, local preferences differ regarding when to combine techniques. A commonly applied rule of thumb among surgeons is to use a 10 cm defect length cutoff to add an FVFG to a massive allograft, whereas in smaller defects an FVFG or allograft is more often selected as a standalone reconstruction. Reversely, in large resections where the defect length exceeds the length of the fibula, standalone massive allografts are often used. Standalone FVFGs are more often used in the upper extremity than in the lower extremity for children. The Capanna technique (and its modifications) combines the advantages of both techniques as it combines a structural allograft with an FVFG, providing the benefits of both initial mechanical stability and biology. This method involves placing a vascularised fibula graft within or adjacent to the cortical bone allograft, possibly leading to lower rates of non-union, fracture, and infection [3,5].

This systematic review describes outcomes of allografts, FVFGs, and the Capanna technique in paediatric patients and young adults following intercalary resection for lower-extremity bone sarcoma. In this review, we specifically assess differences in defect length, union rates, and time to union, the proportion of complications, and time to full weight-bearing (FWB). These insights may contribute to patient-specific decision-making for selecting reconstructive strategies.

## 2. Materials and Methods

### 2.1. Literature Search

Prior to the literature search, a review protocol was developed based on the Preferred Reporting Items for Systematic Reviews (PRISMA) guidelines [6]. The review protocol was prospectively registered on the Open Science Framework (registration under embargo; DOI will be available upon embargo expiry). A literature search was conducted in PubMed, Scopus, Embase, and the Cochrane Library, up to March 2026. The search strategy, developed in collaboration with the institutional medical library, included the terms and synonymous variations in phrasing and spelling “lower extremity”. “tibia” and “femur”, “oncologic” or “sarcoma”, “limb salvage” or “bone reconstruction”, and “allograft” or “Capanna technique” or “vascularised fibula graft”. The full search queries for the different databases are presented in Supplementary S1.

### 2.2. Study Identification and Selection

Studies were included if they reported on the reconstruction of oncologic bone defects in the long bones of the lower extremities using an allograft, an FVFG, or an FVFG in combination with an allograft (Capanna technique or other variants). Only studies addressing intercalary bone defects of the long bones of the lower extremity, following malignant tumour resections in patients younger than 30 years, were included. Intercalary resection involved a circumferential metaphyseal and/or diaphyseal bone segment resection while preserving the proximal and distal joints, followed by reconstruction of the resulting bone defect. Additional inclusion criteria were a minimum follow-up of 12 months and a study population of at least 5 patients. Eligible study designs included randomised controlled trials, cohort studies, case-control studies, and case series. No restrictions were imposed regarding year of publication or language. The full eligibility criteria are summarised in Table 1. Articles that did not meet all the eligibility criteria were still included if data on a subgroup of patients meeting these criteria could be extracted from tables or other available sources.

Studies were excluded if they focused on anatomical regions other than the long bones of the lower extremities or employed other reconstructive methods, such as osteoarticular allografts, arthrodesis, (irradiated or frozen) autograft, double-barreled fibulas, or allograft-prosthetic replacement. Reconstructions following resections for benign tumours, congenital pseudo-arthrosis, or post-traumatic indications were also excluded. Further exclusions included pilot or feasibility studies, animal studies, conference abstracts, and book chapters or reviews.

### 2.3. Screening

All studies were imported into reference software, and duplicates were automatically removed. Screening was conducted independently by two researchers (M.C.C., L.H.M.v.S.) first based on titles and abstracts, and second based on full texts, according to the predefined eligibility criteria. The reasons for exclusion were documented. Conflicting interpretations were resolved by consulting a third reviewer (L.v.d.H.) to reach a consensus.

### 2.4. Data Extraction and Synthesis

Data extraction was independently performed by two reviewers using a standardised data extraction form. Any discrepancies were resolved through discussion and consensus between the reviewers. Demographic data included author, year of publication, years of study, patient demographics (age and sex), tumour histology, reconstruction site (femur or tibia), and follow-up duration. Data regarding outcomes included defect length, time to FWB, time to union, complications and functional outcome, as measured by the Musculoskeletal Tumour Society (MSTS) score, with a range of 0 (poor function) to 30 (optimal limb function) [7]. Defect length was defined as the segment of bone resected due to tumour involvement. The complications extracted included graft fracture, hardware removal, wound dehiscence, infection, delayed union/non-union, amputation, local and distant recurrence, or others. Only data regarding the study population that met the eligibility criteria were extracted and analysed. In studies with mixed populations, when individual patient-level data were available, the mean time to union was recalculated using only the eligible patients. Study-specific mean times to union were subsequently summarised to provide an overall descriptive estimate. Studies were synthesised descriptively and grouped by reconstruction technique and outcomes due to clinical and methodological heterogeneity. Therefore, no meta-analysis, sensitivity analyses, assessment of risk of reporting bias across studies, or certainty of evidence were performed.

### 2.5. Quality Assessment

Study quality was assessed using the Joanna Briggs Institute (JBI) Critical Appraisal Checklist for Case Series [8], which evaluates risk of bias related to confounding, selection, information bias, and reporting quality. Bias assessments were performed in Microsoft Excel (Microsoft Corporation, Redmond, WA, USA, 2023).

## 3. Results

### 3.1. Selection and Inclusion of Studies

**Figure 1:**
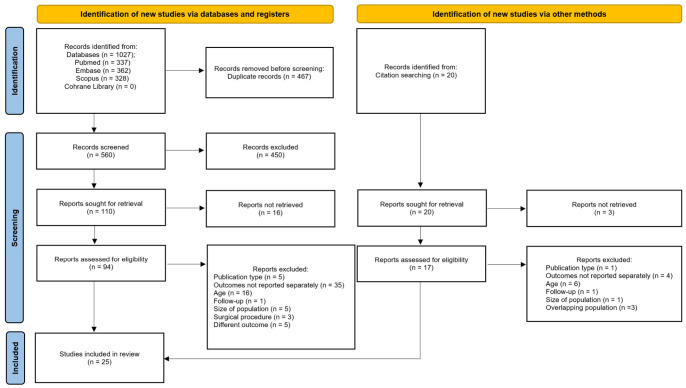
Flow diagram representing the search strategy and results following the Preferred Reporting Items for Systematic Reviews and Meta-Analyses.

In total, 1027 articles were identified through the initial search across PubMed, Scopus, Embase, and the Cochrane Library. Although not included, reviews were the subject of backward citation, and an additional 20 papers were retrieved. After removal of duplicates, title and abstract screening, and full text screening, 25 articles were deemed suitable for descriptive analysis (Figure 1).

### 3.2. Study Characteristics and Description of Participants

The final selection included 23 case series and two cohort studies, published between January 1999 and May 2024. Of these, massive allograft was evaluated in eight studies, FVFG in seven, and the Capanna technique in fourteen; four studies reported outcomes for two reconstruction techniques [4,9,10,11]. The total sample size comprised 391 patients: 147 in the allograft group, 51 in the FVFG group, and 193 in the Capanna group. Fixation was most commonly performed using a plate across studies, while intramedullary nails, screws, or external fixation were seldom used. Mean patient ages were 13.2, 13.2, and 12.0 years, respectively, with female representation of 40.1%, 37.3%, and 35.0%.

Reconstructions were performed in the femur or tibia region; more than half of the patients underwent femoral reconstruction, with two studies focusing exclusively on femoral reconstruction [10,12] and six on tibial reconstruction [9,13,14,15,16,17]. All patients had a malignant bone sarcoma, with osteosarcoma (OS, 63.2%) and Ewing sarcoma (ES, 28.8%) being the most common diagnoses. Mean follow-up was 79.3 months (range: 12–313 months), with 98 months (range 12–288) for allograft, 48 months (range 12–168) for FVFG, and 84.5 months (range 17–313) for the Capanna technique. Study characteristics and the description of participants are summarised in Table 2.

### 3.3. Functional and Postoperative Outcomes

The functional and postoperative outcomes for massive allograft [4,9,10,18,19,20,21,22], the Capanna technique [4,9,10,11,12,13,15,17,23,24,25,26,27,28], and FVFG [11,14,16,29,30,31,32] are summarised separately in Table 3, Table 4 and Table 5.

#### 3.3.1. The Choice of Technique for Reconstruction

In the allograft group, 70.1% (103/147) of patients underwent a reconstruction of the femur, while 29.9% (44/147) underwent a reconstruction of the tibia. In contrast, in the FVFG group, 60.8% (31/51) of patients underwent a reconstruction of the tibia and 39.2% (20/51) of the femur. In the Capanna group, 51.9% (96/185) of patients had a reconstruction of the femur and 48.1% (89/185) of the tibia, although the study of Kapukaya et al. [27] did not specify whether reconstructions were performed on the femur or tibia (4.2%, 8/193).

Regarding defect length, the allograft group showed a mean defect length of 14.9 cm (range: 6–27.5), as reported in six out of eight studies (Table 3). In the Capanna group, the overall mean defect length was 14.7 cm (range: 7–29), as described in twelve out of fourteen studies (Table 4). The FVFG group showed an overall mean defect length of 14.5 cm (range: 6–22), according to six out of seven studies (Table 5).

#### 3.3.2. Time to Union and Full Weight Bearing

In the allograft group, the mean time to union was 8.5 months, as reported in 4 of 8 studies. Misaghi et al. [9] reported 11 ± 2 months, and Houdek et al. [4] reported 10 months (range: 5–20 months), both defining union as bridging callus in three of the four cortices. Moreover, Han et al. [21] reported 9 months (range: 3–21), defining union as the presence of callus or trabecular bone at the allograft–host junctions. Wang et al. [19] reported 2.5 months for the epiphysis and 3.8 months for the diaphysis, assessed during clinical follow-up, although bone union was not explicitly defined. Time to FWB for this group was not reported. Mean MSTS score was 25.8 in 6 of 8 studies (Table 3).

In the FVFG group, the mean time to union was 12.5 months, as reported in 5 of 7 studies. Ruiz-Moya et al. [11] and Khira et al. [14] observed the shortest union times: 4.2 months (range: 4–5 months; union not explicitly defined) and 5.5 ± 1.3 months (defining union as uninterrupted external bony borders between the fibular graft and recipient bone), respectively, while Chen et al. [29] reported 26.2 ± 20.4 months, defining union of the host/allograft junction as bridging callus on three of the four cortices. In addition, Schwarz et al. [16] and Laffosse et al. [30] reported 14.7 ± 13.2 months and 11.9 ± 10.5 months, respectively, with the latter defining union as a bony bridge between the fibular graft and recipient bone with disappearance of the osteotomy line. FWB was achieved after 13.8 months. Mean MSTS score was 25.0, as reported in 4 of 7 studies (Table 5).

In the Capanna group, the mean time to union was 11.4 months, as reported across 6 of 14 studies. Ruiz-Moya et al. [11] reported the shortest mean union time, 7.3 months (range: 3–33), whereas Innocenti et al. [13] reported the longest mean union time of 19.3 months (range: 10–34), assessing it radiographically by the consolidation of the allograft and initial hypertrophy of the vascularised fibula. In all studies, union of fibular graft occurred earlier than union of the allograft. FWB was achieved after a mean time of 15.3 months, as described in 5 of 14 studies. Ten studies reported MSTS scores ≥ 26; the mean MSTS score was 27.6 (Table 4).

#### 3.3.3. Surgical Complications: Graft Fracture, and Non-Union

In the allograft group, the mean fracture proportion was 18.4% (range: 0–45.5%), as reported in all 8 studies. Houdek et al. [4] reported a rate of 45.5% (5 of 11 patients), with three cases requiring revision surgery, whereas Han et al. [21] reported no fractures (0 of 15 patients). Non-union and delayed union data were reported in 6 of 8 studies, with an overall non-union proportion of 13.2%, and delayed union was observed in 4.8% across the allograft group. Infections were reported in 8.2% of patients across all 8 studies.

In the FVFG group, the mean fracture proportion was 40.5% (range: 20–67%), as reported in 5 of 7 studies. Schwarz et al. [16] reported the highest fracture proportion 67% (4 of 6 patients), with all fractures healing after closed reduction, casting, and planned weight bearing. The non-union rate was 14.7% (reported in 5 of 7 studies) and delayed union was observed in 13.6% (4 of 7 studies) across the FVFG group. Infections occurred in 2.7% of patients across 5 of 7 studies.

In the Capanna group, the mean fracture proportion was 23.5% (43/183 patients), with reported rates ranging from 0 to 50% (reported in 13/14 studies). Several studies, including those by Li et al. [25], Kapukaya et al. [27] and Opyrcal et al. [15], reported no fractures. Moran et al. [23] documented two late fractures (>2 years postoperative). Non-union and delayed union rates were reported in 12/14 and 11/14 studies, accounting for 10.3% and 7.9%, respectively, and most studies noted successful primary union. Non-union rates ranged from 0% [9,13,15,23] to 30% [28], with the majority of series reporting values below 20%. In the combined group, 5.7% of patients had infections, reported across 14 of 14 studies.

#### 3.3.4. Disease Recurrence and Limb Salvage

In the allograft group, 16 secondary amputations were reported (16/135, 11.9%): 14 due to recurrence and two due to deep infection, as described in 7 of 8 studies. Most patients received chemotherapy. In the FVFG group, no secondary amputations were reported in 5 of 7 studies. In the Capanna group, 9 secondary amputations were reported (9/175, 5.1%): six due to local recurrence, one due to infection, one due to infection and fracture, and one due to fracture alone. Postoperative complications and sequelae are summarised in Table 3, Table 4 and Table 5.

#### 3.3.5. Quality Assessment

The risk of bias was assessed using the JBI Checklist for Case Series and Cohort Studies and summarised in Supplementary Material S2. Risk of bias was categorised as low (≥80%), moderate (50–79%), or high (<50%). All case series had a low risk of bias, and the two cohort studies had a moderate risk.

## 4. Discussion

This systematic review evaluates three biological reconstruction techniques after intercalary resection for lower-extremity bone sarcoma in paediatric and young adult patients. Original articles on the use of massive allograft, FVFG, and the Capanna technique (i.e., combination of FVFG and massive allograft) are included and assessed for defect length, union rates and time to union, the proportion of complications, and time to full weight-bearing (FWB). These insights may contribute to patient-specific decision-making for selecting appropriate reconstructive strategy.

Across the included studies, FVFG and Capanna technique reconstructions had the longest mean union times (12.5 and 11.4 months), whereas massive allograft reconstructions showed a shorter average time to union (8.5 months). These findings are somewhat surprising given the avascular nature of allografts and their limited biological integration, yet they but confirm massive allograft alone as a reliable reconstructive option [33,34]. The reasons for the longer time to union despite the advantage of intrinsic blood supply that promotes bone formation of the fibular graft could not be determined from the available evidence. Potential biological, mechanical, and/or treatment-related factors that may influence bone healing, such as the use of adjuvant chemotherapy, were not consistently reported, precluding further interpretation. Non-union and delayed union were quite similar between different techniques.

Complication profiles differed across techniques. FVFG was more strongly associated with postoperative fractures (40.5%), versus massive allografts (18.4%) and the Capanna technique (23.5%), reflecting the limited initial load-bearing capacity of the fibula alone, despite its osteogenic potential [35]. Infection rates were lowest for reconstructions with living biology, including FVFG (2.7%) and the Capanna technique (5.7%) versus massive allografts (8.2%). The synergistic combination of mechanical stability from the allograft and biological activity from the fibula likely underpins these findings and support the Capanna technique as a reliable option with low infection risk, particularly in load-bearing bones where mechanical insufficiency is a major concern. This is consistent with previous reports by Bakri et al. and Feltri et al. [36,37] and with long-term results described by Dolen et al. [38].

Functional outcomes including MSTS scores were highest for the Capanna technique (27.6) and a bit lower for FVFG and allograft alone (25 and 25.8, respectively). Time to FWB was only reported for the Capanna technique (mean 15.3 months) and standalone FVFG (mean 13.8 months). Combined with time to union, we feel that mechanical stability, rather than radiographic union alone, determines safe mobilisation [11,35].

There were no differences in mean defect lengths among the different techniques in this review. Traditionally, the Capanna technique is often reserved for larger segmental defects (generally >10 cm). Although we originally wanted to assess differences in techniques used for different defect lengths, and their outcomes, the defect length was often not specified for each individual patient, and therefore firm conclusion cannot be drawn on this rule of thumb cutoff point of 10 cm. Nevertheless, the trend suggests that combined mechanical and biological reconstruction may be beneficial even in smaller gaps when risk factors such as chemotherapy, poor host bone quality, or high mechanical load are present. On the other hand, when defect length exceeds the length of the native fibula, one is confined to the use of massive allograft only. Current evidence suggests that either massive allograft or Capanna technique may be appropriate reconstructive options for intercalary bone defects. The routine use of FVFG as standalone reconstruction may be considered selectively for the youngest patients with protected postoperative rehabilitation, or when allografts are not available to add structural support.

Systemic treatments such as chemotherapy and radiotherapy further affect graft performance by impairing osteogenesis, angiogenesis, and immune defence [39,40]. These effects may disproportionally compromise avascular allografts. Some studies suggest shorter union times in patients not receiving chemotherapy [25,41], but reporting was inconsistent. However, the biological plausibility of systemic treatment impairing bone healing is well established. Surgeons should therefore consider systemic risk factors when selecting a reconstruction strategy, as biologically active techniques may be more resilient under these conditions and Capanna techniques may be particular useful in these patients.

### 4.1. Strengths, Limitations, and Reporting Gaps

This review has several methodological strengths. It is, to our knowledge, the first to systematically compare clinical outcomes of allograft, FVFG, and Capanna reconstructions in paediatric and young adult patients undergoing intercalary resection for lower-extremity bone sarcomas. Previous reviews addressed fewer reconstructive techniques or included broader populations and/or indications [3,5,33,37]. The specific focus on biological reconstruction techniques in this paediatric and young adult population, together with the assessment of clinically meaningful variables including complications, functional outcomes, and reconstruction-related factors such as defect length, enables a comprehensive evaluation of treatment outcomes and may inform clinical decision-making. Adherence to PRISMA guidelines further enhances the robustness and clinical relevance of the findings.

Several limitations, however, must be acknowledged. Study heterogeneity was considerable, particularly in the reporting of definition of union, time to union, functional scores, and complications. Variability in the definition of union and the use of recalculated union times from reported data may limit the comparability of outcomes and should be considered when interpreting the results, as different union criteria may influence reported time-to-union outcomes. Accordingly, differences in patient selection, surgical indications, fixation methods, and outcome reporting further limits direct comparability between reconstruction techniques. Follow-up periods were often limited, potentially underestimating late complications such as graft failure. Details on reoperations, adjuvant therapy, and complication severity were inconsistently reported, introducing reporting bias. The small overall sample size precludes definitive conclusions and the retrospective nature of the included studies may limit the generalisability of the findings, which should be considered when interpreting the reported outcomes. The JBI assessment showed a low risk of bias among case series and moderate risk of bias among the two cohort studies. In addition, the observed shorter union times in allografts may be influenced by confounding factors. Originally, by stratifying outcomes according to defect length, we wanted to explore whether graft choice should be guided by defect length. However, unfortunately defect length was often not specified in the included papers, so this could not be evaluated. Consequently, while the trends observed are consistent and clinically relevant, the strength of evidence remains limited.

### 4.2. Clinical Implications

The findings of this review reaffirm that reconstruction with massive allograft or the Capanna technique are suitable strategies for large segmental reconstructions in children and young adults, particularly in load-bearing bones of the lower extremity [11,42]. The mechanical advantages of adding a massive allograft enables faster and reliable union, earlier weight-bearing, and lower complication rates than FVFG alone. Although standalone FVFG provides biological potential, it suffers from mechanical vulnerability, leading to frequent fractures and delayed mobilisation, even in children, and may not be the ideal reconstruction method in weight bearing localisations (i.e., lower extremities) [35,43]. Capanna reconstructions mitigate these limitations and combine both biology to promote union and mechanical integrity, but this comes at a cost of higher morbidity, longer surgical duration and higher surgical complexity. Massive allografts—other than one might expect—had the shortest time to union and a similar fracture risk in this review. Although this review focused on paediatric and young adult populations, previous studies in adult cohorts have reported higher rates of complications and non-union following biological reconstructions, particularly when using allografts alone [33,37]. The lower rates observed in this review may therefore reflect the enhanced bone regenerative capacity of younger patients, which is related to greater vascularity and osteogenic potential. However, direct comparative analyses between paediatric and adult populations are scarce and would provide valuable insights into the role of skeletal maturity in graft incorporation and complication risk.

For different defect lengths after intercalary resection, evidence is currently insufficient to recommend either technique as a standard approach [11]. Nonetheless, the use of the Capanna technique may be justified in patients with additional risk factors, where improved healing potential and earlier mobilisation could outweigh surgical complexity. In such cases, the decision should be tailored to the individual patient, taking into account defect size, systemic therapy, host bone quality, and anticipated mechanical demands.

Practical considerations must also inform surgical choice. The Capanna technique entails donor-site morbidity, longer operative time, and requires microsurgical expertise, which may not be available in all centres and at all times. Complications such as ankle instability, donor-site pain, and growth disturbances have been reported in younger patients [44]. Cost-effectiveness and resource availability should therefore also guide decision-making.

### 4.3. Recommendations and Future Directions

Further research is needed to refine patient selection for biological reconstructions. Large, multicentre prospective studies are essential to overcome the small sample size and reporting heterogeneity that currently limit the evidence base. Standardised documentation of defect and reconstruction length and outcomes, including definition of union times, standardised reporting on complications, severity, reoperation rates, and functional outcomes, would improve comparability across studies. Moreover, systematic reporting of adjuvant therapies such as chemotherapy and radiotherapy is critical, given their established influence on bone healing and graft integration.

In addition, defect length should be reported not only in absolute terms (cm) but also relative to the overall bone length, as this provides greater clinical relevance, especially in paediatric patients, where the same absolute defect can represent a markedly different anatomical and functional burden. Sanders et al. have shown that relative defect length is a meaningful predictor of long-term outcomes, and adopting this approach should improve the accuracy of treatment planning [45].

Future studies should also aim to define clinical thresholds at which the addition of an FVFG to an allograft becomes beneficial, and hereby evaluate the traditional (unproven) rule of thumb and whether this lies at for example 8, 10, or 12 cm, or at what relative measure. Also, the reporting and evaluating fixation method is of great importance. Reconstructions with compression plates, bridging plates, intramedullary nails or a combination thereof, may also influence time to union and time to weight bearing. In the studies included in this review, the large majority of the patients had plate fixation, but specific details were often not described and outcomes could therefore not be assessed. Alongside structural outcomes, comparative analyses should evaluate cost-effectiveness and donor-site morbidity, particularly in the paediatric population, where complications such as ankle instability, donor-site pain, and growth disturbance are concerns [44]. These considerations are crucial to determine whether the biological and mechanical benefits of the Capanna technique justify its greater surgical complexity and resource requirements.

## 5. Conclusions

The findings of this review suggest that reconstruction with either massive allograft or the Capanna technique are suitable strategies for large segmental bone defects in paediatric and young adult patients after lower-extremity bone sarcoma resection. The mechanical advantages of adding a massive allograft enables faster and reliable union, earlier weight-bearing, and lower complication rates than FVFG alone. Massive allografts demonstrated shorter time to union in this purely paediatric review. Capanna technique may be beneficial when risk factors such as chemotherapy, poor host bone quality, or high mechanical load are present. Until stronger evidence emerges, the reconstruction strategy for children should be tailored to anatomical location, defect size, and patient-specific factors. Future multicentre studies with standardised reporting are essential to establish clear thresholds and optimise patient-tailored reconstruction strategies.

## Figures and Tables

**Table 1:** Inclusion and exclusion criteria.

Inclusion Criteria	Exclusion Criteria
− Intercalary bone defects in long bones of the lower extremities	− Anatomical regions other than long bones of the lower extremities
− Allograft, FVFG or FVFG in combination with allograft (Capanna technique or other variations)	− Intra-articular reconstruction
− Follow-up time ≥ 12 months	− Other reconstruction techniques, such as allograft-prosthesis, osteoarticular allografts, autografts, double-barrel fibula, arthrodesis.
− Resection of primary malignant bone tumours	− Pilot/feasibility studies, animal studies, abstracts only, book chapters, technical remarks, reviews
− RCTs, observational studies or case series with an included population ≥ 5 patients	

**Table 2:** Included study characteristics.

Study	Country	Study Design	Sample Size (*n*)	Reconstruction Technique	Type of Fixation (*n*)	Age (Mean, y)	Females (*n*, %)	Histopathology (*n*—OS, ES, Other)	Follow-Up (Months)
San-Julian et al. [18]	Spain	Case series	14	Allograft	-	7 (2–10)	6 (50)	7, 6, 1	134 (36–204)
Wang et al. [19]	China	Case series	33	Allograft	IMN	12 (8–16)	12 (36)	23, 6, 4	38 (12–72)
Aponte-Tinao et al. [20]	Argentina	Case series	35	Allograft	-	18 (2–50)	16 (46)	35, 0, 0	112 (21–276)
Han et al. [21]	China	Case series	15	Allograft	Plate (9), Nail (5), Plate + Nail (1)	20 (11–29)	1 (7)	15, 0, 0	63 (14–99)
Houdek et al. [4]	USA	Cohort	11	Allograft	Plate (8), IMN + Plate (3)	13 (5–16)	6 (55)	8, 3, 0	156 (36–288)
Kim et al. [22]	South Korea	Case series	12	Allograft	Plate	12 (7–16)	6 (50)	12, 0, 0	67 (31–148)
Misaghi et al. [9]	USA	Case series	6	Allograft	Plate	12 ± 4	4 (67)	2, 2, 2	84 ± 60
Errani et al. [10]	Italy	Cohort	21	Allograft	Plate	12 ± 2	8 (38)	17, 4, 0	130 ± 56
Manfrini et al. [17]	Italy	Case series	10	Combined FVFG-allograft	Plate or screws	11 (7–12)	0 (0)	-	64 (36–101)
Moran et al. [23]	USA	Case series	7	Combined FVFG-allograft	Plate	11 ± 4.8	2 (29)	2, 4, 1	52 (24–84)
Innocenti et al. [13]	Italy	Case series	17	Combined FVFG-allograft	Plate	14 (5–26)	6 (35)	10, 4, 3	135 (28–213)
Li et al. [24]	China	Case series	9	Combined FVFG-allograft	Plate	16 (11–22)	4 (44)	6, 3, 1	34 (17–53)
Li et al. [25]	China	Case series	8	Combined FVFG-allograft	-	11 (9–13)	2 (25)	7, 1, 0	38 (26–53)
Weichman et al. [26]	USA	Case series	10	Combined FVFG-allograft	Plate	14 (3–29)	6 (60)	7, 3, 0	51 (21–104)
Campanacci et al. [12]	Italy	Case series	18	Combined FVFG-allograft	Screw (1), Plate + screw (1), Plate (6)	12 (5–22)	5 (28)	9, 8, 1	131 (24–313)
Houdek et al. [4] ^a^	USA	Cohort	18	Combined FVFG-allograft	Plate	11 (5–16)	9 (50)	10, 5, 3	156 (36–288)
Errani et al. [10] ^a^	Italy	Cohort	25	Combined FVFG-allograft	Plate	11 ± 3	8 (32)	14, 11, 0	117 ± 61
Ruiz-Moya et al. [11] ^b^	Spain	Case series	12	Combined FVFG-allograft	Plate (11), Plate + external fixator (1)	10 (6–13)	6 (50)	8, 4, 0	41 (22–79)
Misaghi et al. [9] ^a^	USA	Case series	11	Combined FVFG-allograft	Plate (10), Plate + IMN (1)	14 ± 5	0 (0)	4, 4, 3	144 ± 72
Kapukaya et al. [27]	Turkey	Case series	8	Combined FVFG-allograft	Plate	11 (7–14)	4 (50)	-	47 (21–115)
Luca et al. [28] ^c^	Italy	Case series	31	Combined FVFG-allograft	Plate	11 (5–17)	9 (29)	10, 12, 1	143 (24–313)
Opyrcal et al. [15]	Poland	Case series	9	Combined FVFG-allograft	-	13 ± 1.7	3 (33)	6, 3, 0	31 (23–51)
Chen et al. [29]	USA	Case series	6	FVFG	Plate	13 (5–27)	3 (50)	2, 3, 1	64 (20–117)
Lafosse et al. [30]	France	Case series	8	FVFG	Plate	13 (11.5–16.5)	2 (25)	3, 5, 0	50 (12–144)
Schwarz et al. [16]	USA	Case series	6	FVFG	Ilizarov frame (3), Plate (3)	12 (6–16)	4 (67)	2, 3, 1	71 (17–168)
Khira et al. [14]	Egypt	Case series	12	FVFG	Screws + external fixation with Ilizarov	18 (14–25)	4 (33)	8, 4, 0	41 (32–52)
Parag et al. [31]	India	Case series	9	FVFG	Plate	19 (9–28)	2 (22)	5, 4, 0	36 (22–74)
Ruiz-Moya et al. [11] ^a,b^	Spain	Case series	5	FVFG	Plate	8 (2–12)	2 (33)	1, 4, 0	51 (39–57)
Karami et al. [32]	Lebanon	Case series	5	FVFG	Plate (4), IMN (1)	10 (7–14)	2 (40)	-	22 (12–48)

Reconstruction techniques: FVFG—free vascularised fibula graft, Allograft combined with FVFG. OS—Osteosarcoma; ES—Ewing’s sarcoma. IMN—intramedullary nail. Reported in mean and standard deviation, median, and range. Age and follow-up are preferably reported as mean and range, but also as mean and standard deviation if the original article provides no information on the range. (^a^) Data derived from different cohorts within the same study. (^b^) The lower-extremity population is separated from the overall patient population. (^c^) Intercalary resection is separated from the interpiphyseal resections.

**Table 3:** Postoperative outcome and complications following massive allograft.

Study	Sample Size (*n*)	Reconstruction Location (*n*)	Defect Length (Mean, cm)	Union Time (Months)	FWB (Months)	MSTS (Mean)	Complications (*n*, %)							LR/Metastases (*n*)
							N = 147	Graft Fracture	Delayed Union; Non-Union	Hardware Removal	Wound Dehiscence	Infection	Amputation	
San-Julian et al. [18]	14	Femur (6), Tibia (8)	13.9 (6–24)	-	-	-	10	3	0, 3	0	0	3	3	3
Wang et al. [19]	33	Femur (24), Tibia (9)	-	3.8 (-)	-	-	4	1	2, 0	1	0	0	3	3
Aponte-Tinao et al. [20]	35	Femur (26), Tibia (9)	-	-	-	26.8 (10–30)	16	11	0, 3	0	0	2	1	3
Han et al. [21]	15	Femur (9), Tibia (6)	13.7 (6–24)	9 (3–21)	-	26 (23–28)	2	0	-, -	2	0	0	1	2
Houdek et al. [4]	11	Femur (7), Tibia (4)	17 (11–26)	10 (5–20)	-	27 (6–30)	9	5	4, -	1	1	2	4	-
Kim et al. [22]	12	Femur (10), Tibia (2)	15.8 (8–27.5)	-	-	28 (24–30)	5	1	0, 3	-	-	2	-	1
Misaghi et al. [9]	6	Femur (0), Tibia (6)	14 ± 4	11 ± 2	-	21 ± 4.2	-	1	-, 1	-	-	2	4	2
Errani et al. [10]	21	Femur (21), Tibia (0)	15 ± 4	-	-	25.9 ± 3.5	12	5	0, 6	3	0	1	0	3

FWB: Full weight bearing. MSTS: Musculoskeletal Tumor Society. MSTS: Musculoskeletal Tumor Society. For Misaghi et al. ([9]), MSTS values were reported as percentages and converted to a 30-point scale by multiplying the reported percentage by 0.3. Reported in mean and standard deviation, median, and range. N = not reported. Age and follow-up are preferably reported as mean and range, but also as mean and standard deviation if the original article provides no information on the range.

**Table 4:** Postoperative outcome and complications following Capanna technique (combined FVFG-allograft).

Study	Sample Size (*n*)	Reconstruction Location (*n*)	Defect Length (Mean, cm)	Union Time (Months)	FWB (Months)	MSTS (Mean)	Complications (*n*, %)							LR/Metastases (*n*)
							N = 193	Graft Fracture	Delayed Union; Non-Union	Hardware Removal	Wound Dehiscence	Infection	Amputation	
Manfrini et al. [18]	10	Femur (0), Tibia (10)	12.5 (10–15)	-	-	28.5 (26–30)	-	-	-, -	-	-	1	1	-
Moran et al. [23]	7	Femur (3), Tibia (4)	13 ± 4.6	9 (-)	-	-	4	2	2, 0	0	0	0	0	-
Innocenti et al. [12]	17	Femur (0), Tibia (17)	15.1 (10–21)	19.3 (10–34)	21.4 (14–36)	27.1 (18–30)	10	5	1, 0	0	4	1	3	4
Li et al. [25]	9	Femur (4), Tibia (5)	12.2 (9–16)	10.9 (8–15)	12.4 (9–16)	27.7 (24–29)	4	0	0, 1	1	1	0	1	4
Li et al. [24]	8	Femur (2), Tibia (6)	-	-	-	26.9 (24–29)	3	1	-, 1	0	0	0	0	1
Weichman et al. [26]	10	Femur (8), Tibia (2)	15.4 (10.5–22)	-	15.4 (9–23)	-	8	3	0, 3	0	1	2	0	1
Campanacci et al. [12]	18	Femur (18), Tibia (0)	18.4 (12–29)	-	-	28.5 (22–30)	12	4	0, 3	4	0	0	0	2
Houdek et al. [4]	18	Femur (8), Tibia (10)	15 (4–30)	11 (5–20)	-	27 (6–30)	-	8	6, -	4	3	0	1	-
Ruiz-Moya et al. [11]	12	Femur (11), Tibia (1)	15.9 (7–22)	7.3 (3–33)	15.1 (9–33)	-	10	6	0, 2	0	0	0	1	3
Misaghi et al. [9]	11	Femur (0), Tibia (11)	14 ± 4	11 ± 5	-	28.2 ± 4.2	-	3	-, 0	-	-	0	0	1
Errani et al. [10]	25	Femur (25), Tibia (0)	18 ± 5	-	-	26.7 ± 2.3	12	7	0, 4	3	0	4	0	8
Kapukaya et al. [27]	8	Femur (-), Tibia (-)	-	-	-	-	6	0	4, 1	0	0	1	0	0
Luca et al. [30]	31	Femur (17), Tibia (14)	14 (9–22)	-	-	28.5 (25.8–30)	14	4	0, 2	0	3	2	2	1
Opyrcal et al. [16]	9	Femur (0), Tibia (9)	12.4 (8–16)	-	12 ± 1.4	27 (23–30)	2	0	0, 0	0	1	0	0	2

FWB: Full weight bearing. MSTS: Musculoskeletal Tumor Society. For Misaghi et al. ([9]), MSTS values were reported as percentages and converted to a 30-point scale by multiplying the reported percentage by 0.3. Reported in mean and standard deviation, median, and range. Age and follow-up are preferably reported as mean and range, but also as mean and standard deviation if the original article provides no information on the range.

**Table 5:** Postoperative outcome and complications following free vascularised fibula graft.

Study	Sample Size (*n*)	Reconstruction Location (*n*)	Defect Length (Mean, cm)	Union Time (Months)	FWB (Months)	MSTS (Mean)	Complications (*n*, %)							LR/Metastases (*n*)
							N = 51	Graft Fracture	Delayed Union; Non-Union	Hardware Removal	Wound Dehiscence	Infection	Amputation	
Chen et al. [29]	6	Femur (1), Tibia (5)	13.2 (11–16)	26.2 (8–60)	-	-	-	-	2, 1	-	-	-	-	-
Lafosse et al. [30]	8	Femur (5), Tibia (3)	-	11.9 (5–45)	11.3 (8–21)	23 (15–29)	-	-	-, -	-	-	-	-	-
Schwarz et al. [16]	6	Femur (0), Tibia (6)	13.8 (9–22)	14.7 (5–40)	15.8 (9–24)	25.8 (19–30)	6	4	1, 2	0	0	0	0	2
Khira et al. [14] *	12	Femur (0), Tibia (12)	14.8 (13–16.5)	5.5 (4.5–8)	-	25.2 (24–27.6)	4	3	-, 0	0	0	1	0	3
Parag et al. [31]	9	Femur (8), Tibia (1)	18.8 (15–22)	-	-	25.9 (24–30)	5	4	-, -	0	0	0	0	1
Ruiz-Moya et al. [11]	5	Femur (2), Tibia (3)	11.6 (6–16.5)	4.2 (4–5)	13.3 (5–18)	-	4	3	0, 1	0	0	0	0	1
Karami et al. [32]	5	Femur (4), Tibia (1)	14.8 (12–19)	-	14.8 (10–25)	-	2	1	0, 1	0	1	0	0	0

FWB: Full weight bearing. MSTS: Musculoskeletal Tumor Society. Reported in mean and standard deviation, median, and range. Age and follow-up are preferably reported as mean and range, but also as mean and standard deviation if the original article provides no information on the range. * Pedicled vascularised fibular grafts.

## Data Availability

The data presented in this study are available on request from the corresponding author. Some of the data may not be publicly available due to confidentially and in accordance with the Dutch Personal Data Protection Act.

## Supplementary Materials

The following supporting information can be downloaded at: https://www.mdpi.com/article/10.3390/children13070952/s1, Supplementary S1: Search queries for all databases searched; Supplementary S2: Quality assessment.

## Abbreviations

The following abbreviations are used in this manuscript:

FVFG	Free vascularised fibular graft
LSS	Limb salvage surgery
FWB	Full weight bearing
PRISMA	Preferred reporting items for systematic reviews
JBI	The Joanna Briggs Institute
ES	Ewing’s sarcoma
OS	Osteosarcoma
MSTS	Musculoskeletal Tumor Society
ORIF	Open reduction and internal fixation
IMN	Intramedullary nail
