# Supplementary material for: Bridging the Gap: Biological Reconstruction with Vascularised Fibula, Massive Allograft, or Capanna Technique After Intercalary Resection in Children and Young Adults with Lower Limb Bone Sarcoma
Source: Children (Basel). 2026 Jul 20;13(7):952. doi: 10.3390/children13070952 (PMC13406841; doi:10.3390/children13070952)
Supplement: Supplementary file 1 [file children-13-00952-s001.zip › Suppl files/Supplementary S1 _ queries_ final.pdf]

Supplementary S1 – Search queries for all databases searched.

A systematic literature search was performed in four electronic databases: PubMed/MEDLINE, Scopus, Embaase, and Web of Science, with the following queries:

- **Pubmed/MEDLINE:** ("Femur"[MeSH Terms] OR "Femur"[Title/Abstract] OR "Tibia"[Title/Abstract] OR "Tibia"[MeSH Terms] OR "Lower Extremity"[Title/Abstract] OR "Lower Extremity"[MeSH Terms] OR "Bones of Lower Extremity"[MeSH Terms]) AND ("Bone Neoplasms"[MeSH Terms] OR "oncolog*"[Title/Abstract] OR "Sarcoma"[MeSH Terms] OR "sarcoma, ewing"[MeSH Terms] OR "Sarcoma"[Title/Abstract]) AND ("Capanna"[Title/Abstract] OR "fibula* graft*"[Title/Abstract] OR "fibula* flap*"[Title/Abstract] OR "free fibula*"[Title/Abstract] OR "Allografts"[MeSH Terms] OR "allograft*"[Title/Abstract] OR "vasculari* fibula*"[Title/Abstract]) AND ("Limb Salvage"[MeSH Terms] OR "bone reconstruction"[Title/Abstract] OR "osseous reconstruction"[Title/Abstract] OR "Limb Salvage"[Title/Abstract])
- **Scopus:** (Femur OR Femur OR Tibia OR Tibia OR "Lower Extremity" OR "Lower Extremity OR "Bones of Lower Extremity") AND ("Bone Neoplasms" OR oncolo* OR Sarcoma OR "sarcoma, ewing" OR Sarcoma) AND (Capanna OR "fibula* graft*" OR "fibula* flap* OR "free fibula*" OR Allografts OR allograft* OR "vasculari* fibula*") AND ("Limb Salvage" OR "bone reconstruction" OR "osseous reconstruction" OR "Limb Salvage")
- **Embase: (**Femur/exp OR Femur:ti,ab OR Tibia:ti,ab OR Tibia/exp OR 'Lower Extremity': ti,ab OR OR 'Lower Extremity'/exp OR 'Bones of Lower Extremity'/exp) AND ('Bone Neoplasms'/exp OR oncolog*:ti,ab OR Sarcoma/exp OR 'sarcoma,ewing'/exp OR Sarcoma:ti ,ab) AND (Capanna:ti,ab OR 'fibula* graft*':ti,ab OR 'fibula* flap*':ti,ab OR 'free fibula*':ti,ab OR Allografts/exp OR allograft*:ti,ab OR 'vasculari* fibula*':ti,ab) AND ('Limb Salvage'/exp OR 'bone reconstruction':ti,ab OR 'osseous reconstruction':ti,ab OR 'Limb Salvage':ti,ab)
- **Cochrane Library:**
  - ID Search
  - #1 ("tibial bone"):ti,ab,kw
  - #2 (femur):ti,ab,kw
  - #3 MeSH descriptor: [Femur] explode all trees
  - #4 MeSH descriptor: [Tibia] explode all trees
  - #5 ("lower extremity"):ti,ab,kw
  - #6 ("lower-limb"):ti,ab,kw
  - #7 #1 OR #2 OR #3 OR #4 OR #5 OR #6
  - #8 MeSH descriptor: [Bone Neoplasms] explode all trees
  - #9 MeSH descriptor: [Sarcoma] explode all trees
  - #10 ("oncology"):ti,ab,kw
  - #11 ("oncologic"):ti,ab,kw
  - #12 (sarcoma):ti,ab,kw
  - #13 #8 OR #9 OR #10 OR #11 OR #12
  - #14 (capanna):ti,ab,kw
  - #15 MeSH descriptor: [Allografts] explode all trees
  - #16 (allograft):ti,ab,kw
  - #17 (vasculariz* NEXT fibula*):ti,ab,kw
  - #18 (fibula* NEXT graft):ti,ab,kw

- #19 (fibula* NEXT flap):ti,ab,kw
- #20 (free NEXT fibula*):ti,ab,kw
- #21 #14 OR #15 OR #16 OR #17 OR #18 OR #19 OR #20
- #22 MeSH descriptor: [Limb Salvage] explode all trees
- #23 (limb salvage):ti,ab,kw
- #24 (bone reconstruction):ti,ab,kw
- #25 (osseous reconstruction):ti,ab,kw
- #26 #22 OR #23 OR #24 OR #25
- #27 #7 AND #13 AND #21 AND #26
