# Supplementary material for: Bridging the Gap: Biological Reconstruction with Vascularised Fibula, Massive Allograft, or Capanna Technique After Intercalary Resection in Children and Young Adults with Lower Limb Bone Sarcoma
Source: Children (Basel). 2026 Jul 20;13(7):952. doi: 10.3390/children13070952 (PMC13406841; doi:10.3390/children13070952)
Supplement: Supplementary file 1 [file children-13-00952-s001.zip › Suppl files/Supplementary S2 _ quality assessment_revised.pdf]

Supplementary S2 – Quality assessment

We present the results of the risk of bias assessment for the included studies using the Joanna Briggs Institute Scales for Case series and Cohort studies. Risk of bias was categorized as low (≥80%), moderate (50–79%), or high (<50%).

	**1.** Were there clear criteria for inclusion in the case series?	**2.** Was the condition measured in a standard, reliable way for all participants included in the case series?	**3.** Were valid methods used for identification of the condition for all participants included in the case series?	**4.** Did the case series have consecutive inclusion of participants?	**5.** Did the case series have complete inclusion of participants?	**6.** Was there clear reporting of the demographics of the participants in the study?	**7.** Was there clear reporting of clinical information of the participants?	**8.** Were the outcomes or follow up results of cases clearly reported?	**9.** Was there clear reporting of the presenting site(s)/clinic(s) demographic information?	**10.** Was statistical analysis appropriate?	**Total Risk of Bias**
**Li J, 2010**	Yes	Yes	Yes	Yes	Yes	Yes	Yes	Yes	No	Yes	Low
**Luca D, 2024**	Yes	Yes	Yes	Yes	Yes	Yes	Yes	Yes	Yes	Yes	Low
**Misaghi A, 2020**	Yes	Yes	Yes	Unclear	Yes	Yes	Yes	Yes	Yes	Yes	Low
**Moran SL, 2006**	Yes	Yes	Yes	Unclear	Unclear	Yes	Yes	Yes	Yes	Unclear	Low
**Oprychal J, 2024**	Yes	Yes	Yes	Yes	Yes	Yes	Yes	Yes	Yes	Yes	Low
**Ruiz-Moya A, 2019**	Yes	Yes	Yes	Unclear	Unclear	Yes	Yes	Yes	Unclear	Yes	Low
**Weichman KE, 2015**	Yes	Unclear	Yes	Yes	Yes	Yes	Yes	Yes	Unclear	Unclear	Low
**Kapukaya R, 2021**	Yes	Yes	Yes	Unclear	Unclear	Yes	Yes	Yes	Yes	Yes	Low
**Wang Z, 2009**	Yes	Yes	Yes	Unclear	Yes	Yes	Yes	Yes	Yes	Yes	Low
**San-Julian M, 2003**	Yes	Yes	Yes	Yes	Yes	Yes	Yes	Yes	Yes	Yes	Low
**Chen CM, 2007**	Yes	Yes	Yes	Yes	No	Yes	Yes	Yes	Yes	Yes	Low
**Li, J 2014**	Yes	Yes	Yes	Yes	Yes	Yes	Yes	Yes	Yes	Yes	Low
**Parag S, 2016**	Yes	Yes	Yes	Unclear	Yes	Yes	Yes	Yes	Yes	Yes	Low

**Han G, 2015**	Yes	Yes	Yes	Unclear	Yes	Yes	Yes	Yes	Yes	Yes	Low
**Aponte-Tinao L, 2015**	Yes	Yes	Yes	Yes	Yes	Yes	Yes	Yes	Yes	Yes	Low
**Campanacci DA, 2018**	Yes	Yes	Yes	Yes	Yes	Yes	Yes	Yes	Yes	Yes	Low
**Manfrini M, 1999**	Yes	Yes	Yes	Unclear	No	Yes	Yes	Yes	Yes	Yes	Low
**Karami RA, 2021**	Yes	Yes	Yes	No	No	Yes	Yes	Yes	Yes	No	Low
**Kim Y, 2020**	Yes	Yes	Yes	Yes	Yes	Yes	Yes	Yes	Yes	Yes	Low
**Khira YM, 2013**	Yes	Yes	Yes	Unclear	Yes	Yes	Yes	Yes	Yes	No	Low
**Innocenti M, 2009**	Yes	Yes	Yes	Unclear	Yes	Yes	Yes	Yes	Yes	Yes	Low
**Schwarz GS, 2012**	Yes	Yes	Yes	Yes	Yes	Yes	Yes	Yes	Yes	Yes	Low
**Laffosse JM, 2007**	Yes	Yes	Yes	Yes	Yes	Yes	Yes	Yes	Yes	Yes	Low

	**1.** Were the two groups similar and recruited from the same population?	**2.** Were the exposures measured similarly to assign people to both exposed and unexposed groups?	**3.** Was the exposure measured in a valid and reliable way?	**4.** Were confounding factors identified?	**5.** Were strategies to deal with confounding factors stated?	**6.** Were the groups/participants free of the outcome at the start of the study (or at the moment of exposure)?	**7.** Were the outcomes measured in a valid and reliable way?	**8.** Was the follow up time reported and sufficient to be long enough for outcomes to occur?	**9.** Was follow up complete, and if not, were the reasons to loss to follow up described and explored?	**10.** Were strategies to address incomplete follow up utilized?	**11.** Was appropriate statistical analysis used?	**Total Risk of Bias**
**Houdek, MT 2018**	Yes	Yes	Yes	No	No	Yes	Yes	Yes	Unclear	No	Yes	Moderate
**Errani, C 2021**	Unclear	Yes	Yes	Yes	No	Yes	Yes	Yes	Uncelar	No	Unclear	Moderate
